# Exploring the Experiences of Cancer Patients Following an Internet‐Based Cognitive Behavioral Therapy for Insomnia With Professional Phone Guidance: The Sleep‐4‐All‐2.0 Study

**DOI:** 10.1002/pon.70532

**Published:** 2026-06-26

**Authors:** Jonathan Journiac, Anne‐Laure Sébert, Louise Zanni, Léonor Fasse, Dominique Hernot, Alexandra Monod, Cécile Charles, Jean‐Bernard Le Provost, Estelle Guerdoux, Guilhem Paillard‐Brunet, Josée Savard, Sarah Dauchy, Diane Boinon

**Affiliations:** ^1^ Department for the Organization of Patient Pathways (DIOPP) Gustave Roussy Villejuif France; ^2^ Laboratoire de Psychopathologie et Processus de Santé (LPPS) Université Paris Cité Boulogne‐Billancourt France; ^3^ Laboratoire de Psychologie clinique Psychopathologie Psychanalyse (PCPP) Université Paris‐Cité Paris France; ^4^ Bordeaux Population Health Université de Bordeaux Bordeaux France; ^5^ Department of Palliative and Supportive Care Psycho‐Oncology Unit Montpellier Cancer Institute (ICM) University of Montpellier Montpellier France; ^6^ Laboratory Epsylon EA4556 University Paul Valéry Montpellier France; ^7^ Psycho‐Oncology Unit Centre Léon Bérard Lyon France; ^8^ École de Psychologie Université Laval Quebec City Québec Canada; ^9^ Centre de recherche du CHU de Québec‐Université Laval et Centre de recherche sur le cancer Université Laval Quebec City Québec Canada; ^10^ Établissement Jeanne Garnier Paris France; ^11^ Centre National Soins Palliatifs Fin de Vie Paris France

**Keywords:** cancer, coping, insomnia, internet‐based cognitive behavioral therapy, patients' recommendations, professional phone guidance, qualitative study

## Abstract

**Background:**

Sleep problems affect 30%–95% of cancer patients. Cognitive Behavioral Therapy for Insomnia is recognized as the most effective treatment for insomnia. However, implementing it is challenging due to a lack of time, human resources, and financial support. Internet‐Based Cognitive Behavioral Therapy (ICBT‐I) is appreciated by patients, but it lacks professional guidance.

**Aims:**

The Sleep‐4‐All‐2.0 study combined a validated ICBT‐I program, lasting 6–12 weeks, with up to four individual, phone‐based ICBT‐I psychology sessions. The current study sought to explore patients' experiences of the program, focusing on their perceptions of the professional guidance.

**Methods:**

Cancer patients from three different hospitals were interviewed. Out of their discourse, a thematic analysis was conducted.

**Results:**

48 patients were included. Three themes were constructed: (1) Coping with the program, (2) Connections at play (professionals, family, patients, sleep interactions), (3) Benefits and limits of psychological guidance. Although most participants were delighted with the program and the guidance, they encountered numerous difficulties due to personal and external issues, as well as the demands of CBT. Participants highlighted the need to be connected to other patients, supported by their families, and to receive well‐informed care from healthcare professionals (HCP). The phone guidance was important for pragmatic and motivational reasons.

**Conclusion:**

In order to improve program follow‐up, clinicians should work beforehand to adjust expectations and take into account the specific vulnerabilities of certain patients.

## 1 Background

Sleep problems affect 30%–95% of cancer patients [1]. Up to half of cancer patients with insomnia receive pharmacological treatments [2], but many patients prefer non‐pharmacological interventions [3].

Cognitive behavioral therapy for insomnia (CBT‐I) is recognized as the most effective treatment for insomnia. It is recommended as the primary treatment, even when associated with a medical condition [4], and is preferred by patients [5]. However, implementing CBT‐I is challenging due to a lack of time, human resources, and financial support [6]. CBT‐I consists of cognitive therapy, sleep restriction, stimulus control, and sleep hygiene. To address these challenges, Savard et al. [7] developed an Internet‐Based Cognitive Behavioral Therapy (ICBT‐I) specifically for cancer patients in Canada, which was later implemented in France [8]. This feasibility study showed that while patients appreciated the intervention, they lacked professional guidance.

The French Sleep‐4‐All‐2.0 multicenter study aimed to address these gaps by combining a validated ICBT‐I program (Insomnet, comprising 6 modules with written information and videos in an animated cartoon format, each lasting between 5 and 20 min) lasting 6–12 weeks with up to four individual phone‐based ICBT‐I psychology sessions (with D.H. or A.M.). These sessions were a guided internet‐based cognitive behavioral therapy, conceptualized as a targeted ‘enhancement’ intervention based on a treatment manual designed for this research to standardize the process as much as possible, rather than as a traditional, full‐scale psychotherapy. Their primary scope was to bridge the gap between self‐guided digital tools and clinical practice by first identifying patient expectations through a motivational interview, which also served to explain the intervention and the use of the web platform. Subsequent calls focused on sustaining motivation, evaluating any difficulties encountered, and personalizing the recommended strategies to facilitate program completion. In particular, the guidance provided by the psychologist aimed to help patients master techniques such as cognitive restructuring, while allowing for the flexible adaptation of behavioral strategies according to the patient's specific symptoms and barriers. Finally, these sessions allowed for a continuous evaluation of how the patient's insomnia had evolved, providing expert guidance in the practical application of behavioral and cognitive strategies throughout the program. At the end of the program, each patient in need of additional help regarding sleep or comorbidities was referred to a specialized, in‐person meeting.

The current study sought to explore patients' experiences, with a focus on their perceptions of the professional guidance.

## 2 Methods

### 2.1 Recruitment of Participants

The Sleep‐4‐All‐2.0 trial was implemented across 3 French cancer centers.

Eligible participants met the following criteria: (1) age ≥ 18 years; (2) a confirmed diagnosis of localized or metastatic cancer; (3) currently undergoing treatment or in the post‐treatment phase; (4) presence of insomnia symptoms, Insomnia Severity Index (ISI) score ≥ 8; (5) sufficient proficiency in reading and understanding French; and (6) ability to use digital tools with ease and to access the internet.

Exclusion criteria included: (1) significant visual, auditory, or cognitive impairments; and (2) current enrollment in another clinical study targeting insomnia treatment.

For the qualitative component of the study, patients were additionally required to have completed the Insomnet program, either in its 6‐week or 12‐week version.

### 2.2 Procedure

When patients were initially enrolled in the Sleep‐4‐All 2.0 study, they were presented with the overall project, both quantitative and qualitative parts. If they agreed to participate, they signed an online informed consent form for both study parts. The entire procedure is described in detail by Boinon et al. [9].

For this part of the study, a male research engineer (J.J.) contacted patients (via text messages and phone calls) 3 months after they had completed the program. The patients did not know J.J. prior to the study. Apart from his title of research engineer, patients had no information regarding J.J.'s characteristics or hypotheses. J.J. is a Rogerian psychologist whose theoretical framework is grounded in CBT, systems theory and health psychology.

Out of the 216 patients who had completed the program, 79 were contacted. Although no one explicitly refused to participate, 27 did not answer at all, 3 initially agreed to participate but did not respond to their phone call by the due date, and 1 declared themselves to be hospitalized. Consequently, our final sample comprises 48 participants (79% of women, mean age = 52, 67% facing breast cancer). Their characteristics are detailed in Tables 1 and 2.

**TABLE 1:** Patients' characteristics.

Genre	*N*	Mean age (years)	Min.‐max. Age (years)	Mean length itw (minutes)	Min.‐max. Length interview	Center	*N*	%
Male	10 (21%)	52	24 – 73	27	16 – 68	Gustave Roussy	28	58
Female	38 (79%)					Léon Bérard	13	27
						Institut du cancer de Montpellier	7	15

**TABLE 2:** Patients' medical characteristics.

Cancer	*n*	%
Breast	32	67
Digestive	3	6
Skin	3	6
Gynecological	2	4
Hematological	2	4
Lung	2	4
Urological	2	4
Endocrine	1	2
Bone metastases	1	2

Patients were contacted to constitute a purposive sample: participants were divided into 4 groups according to the cut‐off of their post‐program ISI score [10] (≥ 8 or < 8) and length of participation in the program (6 or 12 weeks). Each group included 11 to 13 people. This target of approximately 12 patients per group was initially determined based on established guidelines suggesting that thematic saturation is typically reached within relatively homogeneous subgroups of this size [11]. This structure was designed to ensure sufficient information power to explore and represent the diversity of patients' experiences across each subgroup representing a specific treatment trajectory.

### 2.3 Data Collection

Patients who agreed to participate were scheduled for a telephone interview. Interviews were conducted between July 2022 and September 2023. For the researcher, all interviews took place in the home office. Participants were alone and at home, in their cars, or at their workplaces. All semi‐structured interviews were conducted by phone (J.J.) and audio recorded. During interviews, a short presentation of this part of the study was made (see Appendix 1). A dozen questions, developed by the team, untested, were then asked (see Appendix 2). Some of these questions were taken from Sleep‐4‐All‐1. No interviews were repeated. A few field notes were taken during the interviews.

The development of the interview guide was the result of a collaborative effort within the research team, to center participants' subjective accounts of their experience with the online protocol. Participants were asked intentionally broad questions, allowing them to articulate their experiences freely.

### 2.4 Data Analysis

The interviews were conducted and transcribed by J.J., and not returned to the participants. All patients were given pseudonyms.

A thematic analysis was conducted [12], situated within the codebook approach of thematic analysis [13]. This approach was chosen to ensure a structured and systematic analysis of the data through a collaborative team process. The analysis followed a descriptive and inductive orientation, adhering to the COREQ principles [14]. To enhance the reliability of the findings, a double‐blind coding process was implemented by J.J. and A.L.S. A coding framework was developed progressively and refined through iterative discussions among the research team (J.J., A.L.S., and D.B.), all PhD‐level psychologists specialized in psycho‐oncology. These collective meetings aimed to reach an agreement on the definition of codes and the relevance of the significant elements identified. This structured process led to the construction of four main thematic categories summarizing the cancer patients' experiences with the Insomnet program. Results are presented in a tabular format, including illustrative verbatim quotes to ensure transparency and evidence‐based reporting.

### 2.5 Ethics Approval

The Sleep‐4‐All 2.0 study was approved by the French Ethics Committee (N° ID RCB: 2021‐A01891‐40). All participants were first informed orally and then given time to consider and, if they wished, signed a written informed consent form.

## 3 Results

### 3.1 Themes

Themes regarding patients' experiences of the program, identifying their difficulties, exploring their recommendations, how they perceived their interactions because of sleep difficulties and cancer, and how they viewed the provided psychological guidance, were constructed.

Three themes are presented here:

1. Coping with the program
2. Connections at play
3. Benefits and limits of the psychological guidance

A fourth theme, regarding patients' recommendations, was constructed and is available as a supplemental file. The themes, sub‐themes, and verbatim are detailed in Table 3, 4, 5.

**TABLE 3:** Theme 1—Coping with the program.

Difficulties	Physiological and psychological factors	Duration of insomnia	*Annie: Since the end of treatment, I've had problems, severe sleep problems, but they were problems that were, um… Well, they may have been exacerbated by the illness, but they've been around for a very long time.*
		Disease, treatments, and pain	*Stéphane: No, because most of what keeps me awake is the pain. So as long as I'm in pain, I can take any program in the world. When you're crying and screaming in pain, well, you can't sleep.*
			*Monique: There were mornings when I was uh… It was a bit complicated because with the chemo, I couldn't concentrate on the computer.*
			*Chantal: There was a whole period when I couldn't follow the program properly because I was undergoing radiotherapy.*
			*Chantal: There are things it's not so easy to put in place, so as not to re‐emphasize the illness. Because somewhere along the line, it's linked to that, and um, there comes a time when you want to move on to something else.*
		Many forms of anxiety	*Sylvie: I've had a bit of a complicated year. (…) At times, I have the impression that the little devils are stronger than we are, and it's harder (…) You quickly think about many things, and the negative often takes over… You know what I mean, that side… That was the hardest part.*
			*Catherine: When I'm in that phase where I'm anxious, where I'm clearly having anxiety attacks.*
			*Sylvie: These negative thoughts that get in the way, I found it hard at times to let them go. (…) And you may well say to yourself, “come on, think differently”, (…) in the end, you have to be very strong, even psychologically, and that's what can be disturbing*
			*Colette: It's more like… Like, anticipating things that don't exist. You know what I mean?*
			*Catherine: And when I'm in a phase of insomnia, with dark thoughts in my head, well, it's complicated to change them at that moment.*
			*Olivier: Performance anxiety! That's it. Saying to yourself, “Well, I shouldn't wake up”, bingo, I woke up.*
			*Françoise: I always want to do everything just as I'm told, and not being able to apply what I was told made me feel a bit guilty because then I'd say to myself, “Well, you see, you can't sleep, you just have to apply what you're told”.*
			*Nadine: I was a little afraid to go to bed, I had death on my mind. And so it really upset me.*
	External factors	Material conditions	*Audrey: I'm in a boarding school in France, (…) I have a* *7* *m* ^*2*^ *room to sleep in, so getting up is complicated (laughs). And then when I go home, I'm not all alone, so it's also complicated to… Actually, it's more a question of material problems than motivation, you know?*
			*Emma: After that, I had a bit of difficulty with my schedule, I didn't have a regular rhythm,* *etc*.*, so it was a bit complicated for me sometimes to apply the advice.*
			*Audrey: I'm currently at school for 2 years. And I change places quite often, so uh… (laughs). It's not very conducive to… To ritualizing a bit the… Sleep.*
		Holidays	*Valérie: If I'd known (…) I might have done it at a time when I'm not going on vacation, when I'm in my usual rhythm, so to speak. It would have been easier to apply.*
			*Stéphane: with the time difference when I was on vacation, it was complicated.*
			*Emma: One of the pieces of advice was to, well, get up at a fixed time,* *etc*. *Even if you haven't slept, get up anyway and go about your day and so on, at the same times. (Laughs) It wasn't feasible.*
		Work	*Patrick: I think I should have been… Less involved with other things. Less solicited by my work (…) less solicited by my family (…) I found it hard to make this… This thing here, this treatment, compatible. This part of the treatment with… Uh my, my life, my everyday life.*
			*Elodie: I had a short period where things weren't going so well, but I think it's because I went back to work…*
		Family life	*Isabelle: I find it hard to get up in the middle of the night, I've got my family asleep and I'm thinking what… I'm going to wake them up*
			*Chantal: I have to admit I understood the sleep restriction, so I implemented it. But it really required a real effort for me in the sense that you isolate yourself from the family rhythm you're in.*
			*Catherine: What's complicated is getting up, because I've got a dog, so when… If I'm not asleep and I get up, the dog barks and wakes everyone up, well no, it's… (Laughs) It's complicated. (Laughs) It's complicated.*
	The program	Unrealistic expectations	*Hélène: Well, I was hoping that it would magically solve all my sleep problems. (Laughs)*
		Starting off	*François: It was a bit abstract. At first, you don't know where you're going.*
		Staying up late	*Annie: Sometimes I'd stay until eleven, eleven‐thirty, just to stay up late. At the beginning, it was very, very complicated for me because I was so tired.*
			*Françoise: It was complicated to put in place really, sleeping three and a half hours a night, I couldn't go to bed at three in the morning to get up at half past six, it was just too much. Even if I wanted to… I understood the principle that you had to be really, really tired to go to bed and all that, but it wasn't applicable. The next morning, when I had to get up, I just couldn't do it.*
		Avoiding naps	*Sylviane: I still took naps, because I was very tired from my cancer.*
		Avoiding coffee	Mireille: It's true that I'm a bit sleepier in the afternoon, I'm a bit more… Er a bit more… I could do with a little… Well yeah I, I enjoy my coffee and I miss it a bit.
		No screen	*Julie: No screen uh… Before going to sleep, I admit uh… A bit complicated for me.*
		Television off the bedroom	*Camille: There are things, for example, that were recommended to me in the videos that I tried to apply, but in the end were so ingrained in my way of life that I didn't manage to establish them to the end. But I realized that in the end it had no impact on the fact that I wasn't sleeping. For example: I have a TV in my bedroom.*
		Not reading in bed	*Marie‐Thérèse: Something very complicated for me, not reading in bed before going to sleep, before trying to fall asleep. That's where it was more complicated for me.*
		Getting out of bed at night	Isabelle: I have a hard time with that, if I don't sleep at night, well, I stay [in bed] anyway…
			*Audrey: Except getting up when you can't sleep at night, except getting up, changing rooms, doing something else, reading a book* *etc*. *And going back to bed when you're really tired. That's the only thing I haven't been able to do.*
		Sleep calendar	*Nadine: The sleep calendar, so that was quite restrictive because… It's true that at first I did it in the evening, but I couldn't necessarily remember what time I'd fallen asleep, how many times I'd woken up and couldn't get back to sleep. So the questions were very precise, and every day was very demanding. Sometimes I didn't have time to fill it*
		Cognitive restructuring	*Marine: I had a hard time grasping that part of it, that grid uh… I didn't necessarily use it. It helped me to ask myself questions about other things, but uh voilà, I didn't necessarily uh… I, I… I'm having trouble finding the point of this, this thing.*
			*Marine: I think that part about automatic thoughts, well… In fact, they were concepts that, er… I don't know, maybe they're concepts that we're not used to. I had to go over it again and again to get it to speak to me (…) Anyway, that part was really hard for me to get my head around. I had, I had trouble understanding it.*
			*Marine: Uh, to understand what, what… What were these mechanisms and uh, and when I could have these thoughts. I couldn't really see what… Yeah, I'm having trouble seeing it.*
			Catherine: Cognitive restructuring, that's still complicated for me. I need to reread this module again, which was quite long, by the way.
			*Karine: In terms of content, automatic thoughts are a little more complicated to set up, they require… After that, it's not something you can do on your own but, um… But at the beginning, it's true that it's a little, a little complicated to… To grasp.*
			*Véronique: I'd had a phone call from the psychologist. Talking about it again, talking about the subject of sleep deprivation, let's just say that it reactivated the idea of saying to myself after the following nights, “Will I sleep well?” Basically, sleep was a bit of a taboo subject, the one not to talk about. And I realize that the less I talked about it after that, the better it went. (…) The first time we had a little check‐up, it had been difficult after falling asleep, sleep had been disturbed again.*
Strategies	Strategies to facilitate applying the rules	Get moving so you don't fall asleep too early	*Ginette: I used to fall asleep a lot in front of the TV (…) Well, I avoid, when I feel sleep coming on, well I, I used to sit down instead of lying on my sofa. I'd sit down to avoid falling asleep at nine, nine‐thirty and uh… Ruining uh, ruining my, my uh, my main moment of falling asleep.*
		Set an alarm to remind you to turn off the phone	*Laure: I've forgotten my phone a few times, I said be careful, now we're going to set an alarm to make sure we put it in airplane mode, turn it off and, or put it somewhere else.*
		Keeping busy at night (work, easy reading, podcasts)	*Chantal: So I've tried, I've tried crochet, I've tried, the, the cross‐stitch. Uh… But what really suits me, honestly, is reading. Then I had to figure out the strategy. (…) But I had to select, maybe during the day, small newspaper articles, things without stakes where, uh, even if I start again, it's not too serious, but it doesn't jeopardize, well, in fact the overall understanding of the whole book.*
			*Sophie: So sometimes it happens to me when I've really got something to do or when I've got a file to finish. I say to myself, well, I'll… If I can't sleep, I'll at least make some progress on this file, so that tomorrow I'll be able to sleep later*
			*Maurice: If I wake up in the middle of the night and I'm already agitated, preoccupied by a thought, then it's more difficult. So that's where I got the habit, but it's been a long time… (…) So it's a stratagem I'm a bit addicted to, which is that every night when I go to sleep I make sure I have a little iPhone on my bedside table with A voix nue or programs like that of interviews always calm things. If I start listening to something hectic or an adventure story, it'll wake me up and I'll have to start all over again (laughs).*
		Appealing to reason, understanding and logic	*Julie: When you can't sleep, just get out of bed. That, that chapter was hard to get through. (Laughs) That one was hard to get through. But once you've got through it, well, actually, uh, it makes sense. And once it seems logical, well… Well, we apply it a lot better and um, well, the results are there.*
			*Mireille: It's true that I'm a bit sleepier in the afternoon, I'm a bit more… Er a bit more… I could do with a little… Well yeah, I, I enjoy my coffee and I kind of miss it. (…) Then I tell myself it's for my well‐being too, it helps me sleep better at night.*
			*Julie: Uh well at the beginning, necessarily uh… No …. No screen uh… Before going to sleep, I admit uh… A bit complicated for me. But it's the same, it seems logical, since you have to put your brain to rest. In fact, it's once you've understood the process of why you're doing it. In fact, it seems so logical*
	Getting to grips with the sleep diary		*Olivier: The idea is to compare yourself to yourself, so basically, did you sleep more than the night before or less? Even if I'm wrong, I don't know, by a quarter of an hour every day, in the end it's not a big deal.*
			*René: I forced myself to do it every day because I said to myself, if you don't do it one day, tomorrow you're going to say to yourself, wait a minute, was it that night or the night before? So every day I'd get up, have breakfast and then go straight to my screen or my smartphone.*
	Cognitive restructuring	Repeated the automatic thoughts module several times to fully understand it	*Karine: I've had to redo this module several times to get a good understanding of how to set up automatic thoughts.*
		Paper support to print out helps with cognitive restructuring	*Karine: Being able to have a table and a grid of… To have it on paper, it also helps when you don't necessarily have access to the site all the time. And then, uh, to capture… To capture your thoughts, it's, it's not bad.*
		Focus on breathing rather than thoughts	*Olivier: To avoid paying attention to thoughts, that's how I… Concentrating on my breathing, for example, works well. And I find that I go back to sleep. (…) Don't focus on thoughts (…) Even if they're here, don't pay attention to them, but focus on something else, like breathing for example.*
		When thoughts are difficult to change, get up and do something physical	*Dominique: if I, I don't go back to sleep right away, if, if it's complicated at the thought level to… To move the thought, well even if it, it will move but… Well I'll go and do things physically uh…Yes. And then it gets better.*
		Clearing up negative thoughts	*Amélie: I'll try to get around it (…) by saying [to myself] you're getting ideas in your head, you don't know exactly what happened, why it happened like that. So try to see the positive side and stop it.*
		Promoting positive thinking through pleasure	*Sylvie: I still try, when sleep is falling, to put on things I like or read things I like to try and encourage, to take away a bit of the negative and put on a bit of the positive*
		Repeating “I'm not thinking about anything” over and over again	*Valérie: He kept repeating to himself: “I think of nothing, I think of nothing, I think of nothing”. And I sometimes use that. So I know it's not part of the program. (Laughs) I've found that it works very, very well, because repeating it over and over, well, it doesn't leave the… It doesn't leave room for any other idea to, to come and… To ruin our sleep, well, our wakefulness, at night*
	Going back to the program	Readjusting to the program when sleep is lacking	*Laure: And then I thought: “Ah, ah…. Hop, hop (laughs), I'll pick things up right away”. Because I didn't want to fall back into the same old habits. In fact, it's all about readjustments. It's constantly thinking about how to readjust before we fall too far into things.*
		Downloaded the videos, watch them again if necessary	*Elodie: The person I'd had, uh, in follow‐up, uh, had told me that I could, uh, download the videos, so I got them on my computer. So I could watch them again if one day I had problems sleeping, to watch a bit of the, the… The tips.*
		Sheet of paper next to bed on sleep rules	*Elodie: I'd made myself a sheet which is, which is stuck next to my bed there, on the rules of sleep.*
		Made her own summary to dive back into the program	*Sylviane: I even, I even made a summary for myself, (…) because I tell myself that from time to time so as not to lose elements*
		Printed the summaries and reread them again	*Alain: The, the summaries are extremely well done. So well done, in fact, that I've printed them out from time to time. I still, I still reread them.*
Remaining difficulties	Physiological difficulties	Persistent sleep difficulties	*Annie: I don't have any problems in my life, and yet I don't sleep.*
		Treatments prevent from sleeping	*Amélie: The pain is always there. (…) Right now, while I'm undergoing chemo, the pain is extreme.*
		Pain	*Stéphane: So the problem isn't the program, it's the untreated pain. (…) So [the problem] is more the disease than the program.*
		Fatigue	*Sophie: I'm less tired than when I started the program. Then I'm still, I'm still pretty tired, but it's better.*
	Behavioral difficulties	Too difficult to get up at night	*Sophie: The one I haven't managed to tame so far is saying that when you wake up and you've been tossing and turning in bed for over half an hour, you've got to get up. That's (laughs)… I just can't do it. (…) I actually find it very guilt‐inducing to get up.*
	Mood and cognitive difficulties	Invading thoughts	*Sylvie: You can say to yourself: “come on, be positive, you're strong, there's no reason for it”, but in fact you realize that at night it knocks you down (…). This little “devilish” voice, (…) this silence, it always encourages these thoughts and that's harder, trying to find something positive, it's complicated at that moment.*
		Giving up on cognitive restructuring	*Maurice: The presentation you made on your video montage, on harmful thoughts and all that, the way you kind of rethink things, try to think differently, well, that's nice, but when you've really got something on your mind, it's immediately limiting, it's a bit ideal. (…) Because I don't see much of a solution, to the point where I've given up trying to unravel what was on display by doing this, by doing that. I don't really believe in it myself, maybe wrongly. Maybe I didn't try hard enough, I don't know.*
		Being treated for trauma	*Amélie: I'm being treated for trauma, so it's due to certain reflexes I have that prevent me from sleeping, and now I'm undergoing treatment for therapy.*
Post‐program needs	Improving sleep with or without a “trick”	Quitting medicine	*Hélène: But I haven't completely, completely stopped [the anxyiolytics], and that's one of my goals too, because when I'm stressed, it's the thing that helps me sleep.*
		Avoiding having to listen to something to fall asleep	*Maurice: it always annoys me to be a bit dependent on having a little something to listen to go back to sleep more easily, but that's the drawback, that's the thing I haven't managed to solve*
		Identifying sleep signals	*Nadine: Sometimes I'm fooled, I'll doze off on the sofa and then I'll think: “Oh dear, I, I felt sleep coming on, I should have…”. So sometimes I'm fooled and then I wake up after an hour on the sofa, I'm not in my bed. So that was another thing I had to sort out. It's all about understanding sleep signals.*
	Calling on specialists	Sleep clinic	Patrick: Maybe come and spend a night, film it and then watch what happens together afterward, like they do in sleep clinics.
		Managing side effects	*Camille: And then I think I'm going to redirect myself toward a sophrologist or someone, I haven't decided on the method yet, to try and see how I can untangle all that [the side effects].*
	Maintaining the program	Post‐program access	*Isabelle: Do I still have access to the modules if I want to listen to them again? Because I think I also need to listen to them again, to get the whole thing back into my head.*
		Program summary	*Laure: There's just one thing that's a shame: we don't have a sort of little booklet, because then all the… With all the, all the tips, everything that's been set up and that was on the program. Because… I find that it's… It would have… It would have helped me a lot in fact (laughs). Even to continue, I think it's uh… It's important.*
		Excel file to calculate off‐program sleep efficiency	*Elodie: I'm going to start making myself an Excel spreadsheet, in fact, to transcribe a little bit the, the things, the, the requests so that I can calculate my efficiency.*
	Deepening cognitive and psychological work	Doing more cognitive restructuring	*Laure: Sometimes we can be irritated by something and we go back to the facts in a very factual way, and we… We go back to our… At the level of emotions uh… To readjust and say to ourselves, well in fact, I… I got angry or I was very angry, and I was in a stressful situation over something that didn't deserve it actually. (…) That I don't even do, but that on the other hand, yeah I find it's the thing that I… That I would… That I should do in fact. It would be good… in my… A bit in my routine.*
		Reviewing cognitive biases	*Annie: I've got to go over the cognitive bias stuff again. I mean, sometimes I say to myself, I've got to go back, maybe read things again, especially about cognitive biases*
		Starting psychotherapy	*Martine: All these problems don't just come from sleep, they don't just come from cancer. It's all intertwined. But sincerely, the fact that you see, I have tears in my eyes talking to you about sleep, means that I need [psychotherapy], yes.*

**TABLE 4:** Theme 2—Connections at play.

Healthcare relationships	Appreciated care	Quality hospital	*Catherine: And the fact that it's in a hospital, that it's Gustave Roussy that's offering it, well… Well, for me, it was a guarantee of great seriousness. And in a way, to avoid charlatan methods.*
		Gratitude	*Stéphane: I just wanted to sincerely thank you and the people who work on this kind of project (…). It's not necessarily rewarding, but it's super useful because you're making progress in… people's well‐being. So I really wanted to thank you for your work, and especially the teams.*
	Disrupted care pathway	Misunderstanding from GP	*Catherine: I've also seen a GP: “Well, in my case, when I have an insomnia attack, I'm afraid to go to bed”. And he said: “No, but that's not possible, I'm just waiting for that, it's time to go to bed”. Well, I've also come up against doctors who don't understand this.*
		Abandonment after treatment	*Valérie: After the illness (…) you already feel a bit let down by the doctors (…). Once the treatments are over, it's true that it's poof! There's nothing left and you feel a bit empty. And for me, it was reassuring to still have contact with the hospital environment.*
	Information issues	Lack of time and information from oncologists	*Catherine: Doctors are really in a hurry! They do their job really well, but… Unfortunately, they don't have enough time to inform people about what's going on outside of their area of expertise. So (…) there are lots of things on offer at Gustave Roussy, but if the patient doesn't take the trouble to find out what's available…*
Family ties	Family impact of the program	Fear of disturbance	*Chantal: I have to admit that I understood the sleep restriction, so I implemented it. But it really required a real effort for me in the sense that you isolate yourself from the family rhythm you're in. I had to make the decision, and what's more, I had to explain it to the people around me, et caetera. Particularly my husband, who's extremely understanding! It wasn't a problem. (…) I didn't want to disturb him when I went to bed for fear of waking him up, you know? Because I preferred, uh, getting up early in the morning, because that's what he does, so I tried to find a time when he wouldn't mind waking up. So to enforce the sleep restriction, I went to bed much, much later. (…) When you don't live alone or when, uh… well, there's an impact.*
	Support	Help to go to bed on time	*Ginette: I was away for (…) several weeks with the family and they all told me to go to bed when I had to because they knew that if I stayed up with them until midnight I'd have an impossible night.*
		Husband came to see her at night	*Marie‐Thérèse: [At night] I wasn't all alone in my corner, brooding, trying to sleep, and no, no. I was well supported by my husband.*
		Program followed by the husband	*Marie‐Thérèse: To make things go more smoothly, as my husband is also retired, well, he's staying with me, that's all (laughs). So we've almost done the program together. He doesn't have any sleeping problems, but let's just say that we've almost done the program together, because I need to be able to keep to the hours when… That I said I would, uh… Uh… Well, listen, to help me out, he stayed with me.*
	Including them	Passing on the rules to teenagers	*Sophie: My teenagers sleep very badly and there are a few… There are a few rules that I make them apply. Yes, there are little things that I pass on.*
		Trying to convince the husband	*Ginette: Since my husband naps all the time, I tried to tell him to take shorter naps, but he didn't really follow me on the subject.*
	Distance from family	Different schedules	*Nathalie: If I don't feel like sleeping, I'm not going to… Even if the whole family, my little family, goes to bed. Well, too bad, I'll stay up (laughs). I'm not going to force myself to go to bed. I'm… I'm waiting.*
		Culpabilisation	*Sophie: I've always slept late and worked a lot… And the number of times people have said to me: “No wonder you've got cancer”.*
Peer interactions	Meeting them	Feeling lonely	*Nadine: I think that's what I've been missing since I've been ill (…) it's communication with people who are ill too, so that we can give each other tips and support each other. And I'm a bit alone when it comes to illness.*
	Promoting the program	Would recommend though unsatisfied	*Françoise: It can certainly help a lot of people, it's still something I'd recommend, even if it didn't bring me quite what I expected, but I'd still recommend it to people, if I had people who told me they couldn't sleep and that they had cancer, I'd still recommend it.*
		Asking insomniac friends	*Sylviane: I can talk to friends who say to me: “Well, I've got insomnia”. I say, for example: “Do you have a healthy lifestyle? Do you do that? Do you have a sleep hygiene? Do you do that? Do you know what automatic thoughts are?”.*
		Involving other professionals	*Catherine: In supportive care, there's an image consultant (…) who gives lots of advice about the disease (…) well, she could inform people about what you're doing. Like the socio‐aesthetician too, (…) I don't know if they know about your program, but they meet lots of women too.*
		Access for everyone	*Agnès: It should not be limited to people with cancer, because so many people have sleep problems. So it would be good for it to be promoted by doctors, in fact even by general practitioners.*
		Patients as best intermediaries	*Chantal: I don't know how you're going to open it up and how you're going to make it evolve. But patients, I think, are your best intermediaries.*
Reconceptualizing sleep	A new way to look at sleep	No more fear	*Maurice: I'm no longer afraid of sleep and I'm managing it much better.*
		No more fighting	*Agnès: I was leaving to get on the sleep train and fall asleep, and not at all to do an all‐night fight.*
		Sleeping more is not sleeping better	*Laure: It wasn't because I was going to sleep more that I was going to sleep better, that I was going to get more quality sleep and so on.*
		A more objective view of sleep	*Chantal: From time to time, there were moments when I'd say to myself: “Ha! I slept really badly”. And in fact my sleep efficiency wasn't bad at all!*
		Overhaul of preconceived ideas	*Catherine: A complete overhaul of the preconceived, false preconceived ideas we have about sleep.*
	Acceptance	A subject but not a problem	*Audrey: It's a subject, not a problem. (…) Well, a problem is: (…) “Oh dear, I haven't slept enough! Oh dear, I'm tired! Oh dear, what am I going to do?” You see, we're trying to find solutions to a problem. Whereas a subject is: “I didn't sleep well again (laughs), but it doesn't matter, my day will still go well, and then I'll have a better night later”.*
		Keeping things in perspective	*Dominique: When I wake up, I say to myself: well, it's not a big deal, you're going to do something else, you're going to work on something else (…) I'm much more, uh, relaxed. (Laughter)*
	Less anxiety	Stopping comparisons with before	*Françoise: I don't sleep like I used to, that's for sure, but in any case I've understood that now I shouldn't compare things with before (…) so I take it one day at a time and that's it.*
		No more brooding	*Camille: I don't have those bedtime brooding anymore.*
		Getting rid of a negative bias	*Annie: Getting into this program structured my ideas. It took me away from the bad appraisals I could have had about the fact that “here I am, not sleeping, I'm going to have a rotten day”. Uh, no, not necessarily, because when I said that, I realized that there are times when I feel like I'm not sleeping, and the next day I manage to have a day, I live normally, I work normally, there you go, so that's already the negative bias I've taken away.*
		Seeing difficulties as normal	*Ginette: It's not all about having been ill or being old. There are also things that are, um, normal. And uh, it's also good to remind yourself of this, because it doesn't confuse your mind too much when you, when you try to uh… When you go to bed and you have to sleep. That helped me too. The study helped me with that.*
	Being nicer to yourself	Being less demanding	*Martine: What has it done for me personally? Well, I repeat, it's easy to say, that maybe you need to be less…. Less demanding of yourself.*
		No more guilt	*Sophie: It took a lot of the guilt away. Because the day I was going to decide to get up and do something else, I knew it wasn't bad.*

**TABLE 5:** Theme 3—Benefits and limits of psychological guidance.

A human relationship	A pleasant person	Lovely person	*Amélie: It made me really happy because she was lovely, and whenever I had doubts or felt anxious about my trip, she listened to me. It was also important for me to be able to share my feelings and impressions in the moment.*
		Available	*Michel: The fact that she was available—I never needed to contact her outside of those appointments, but in any case, I knew it was possible. Um, that too, it was, it's very helpful.*
		Reassuring	*Sylvie: It just reassures me to know that I have someone. If something goes wrong, there you go. (…) Whether we call each other or not, it's important to know that I have someone and that it matters to me. There's good follow‐up, because that's also important, knowing that you're being helped, that you're being listened to, that it's something…*
	A human connection	Human ear	*Agnès: It did allow us to have a human being, at some point, to review the situation, rather than always being faced with these videos, so… There's a human ear to listen at some point, which is nice, anyway. (Laughter)*
		A welcome outside perspective	*Alain: I liked it because it's also good in relation to his personal experience with an outside perspective. It's a bit of a positive side, it's the outside perspective (…) with a bit of hindsight.*
		Not feeling lonely	*Annie: The sessions with the psychologist were a bit like a relay point, let's say, during the 12 weeks, so that was good because you don't feel alone behind your screen watching videos.*
Numerous roles	Providing a framework	A regular relationship	*Michel: It was really, really good. And the fact that we had a regular relationship, which was already somewhat imposed by the schedule.*
		Setting up the program	*Julie: The explanation at the beginning is really important, though, in order to be able to implement the program properly.*
		(Re)defining goals and expectations	*Amélie: As the program progressed, I realized that my goals were actually wrong and unattainable, and that with the help of the calls, I was able to redefine coherent goals and, above all, expectations.*
		Reviewing the situation	*Patrick: But even if she hadn't made any useful comments, there was still the opportunity to review the situation, which was, um… Something I always found, and still find, useful.*
	Pedagogy	Clarifying	*Karine: She explained a few things to me again, specifically about automatic thoughts, which were a little… That I had just done and which were a little complicated to implement. So she explained a few points about that to me again.*
		Teaching how to use the tools	*Alain: On the one hand, there's the toolbox, your own experience, and then, well, interacting with the person is kind of how you use them.*
		Explaining potential benefits	*Sandrine: I was hesitant because I was apprehensive about implementing them, but she explained why it would be beneficial.*
	Adaptation	Suggesting adaptations	*Sylviane: Yeah, that's what I told the therapist. I said, “Listen, here's the thing…” She said, “No, no, just do it at your own pace.” And, um… Well, it's working really, really well, actually.*
		Referring to another psychologist	*Annie: She suggested I also see the psychologist in Chevilly, “if you need to”.*
Supportive guidance	Valuable	Appreciated	*Marie‐Thérèse: I thought it was very good. I really appreciated the follow‐up.*
		Effective	*Sylviane: We have effective support from a psychologist.*
		Useful	*Patrick: It was always useful. It helped me to verbalize, to put into words what was happening. And it was also a way of reviewing progress or lack thereof, depending on the phone calls.*
	Cognitions	Motivation	*Stéphanie: It helped me to do it regularly. Knowing that I'm going to have interviews where I'll have to talk about it,* *etc*. *So, it's an incentive to follow the program with attention. And then, um, and then it's encouraging.*
		Raising new questions	*Marine: I thought it was good that there was interaction, because we were asked questions, and that allowed me to focus a little bit on asking myself questions that I might not necessarily have asked myself otherwise.*
		Accepting recovery	*Catherine: I was still on sick leave and I was having a hard time accepting the recovery period. So she really helped me to accept it.*
	Emotions	Reframing performance anxiety	*Olivier: I was thinking, “Oh dear, this is silly… It's annoying, I keep waking up at night,”* *etc*. *And she said to me, “Yes, but in the end it's not a big deal”, and that's it, to be careful with performance anxiety. So let's say it allows you to reframe things a little bit.*
		Stop feeling guilty	*Valérie: And she helped me to stop feeling guilty about not taking everything on board, not taking all the advice, and not applying everything.*
A limited support	Pointless	Not necessary	*Céline: I think I could have managed just fine with the online platform alone.*
		Not helpful	*Emma: I didn't see much help, for me anyway.*
		Duplicate	*Christine: For me it wasn't, so to speak, a huge support, because I was already being treated by someone else.*
	Too entrenched in research	Too technical	*Jean‐Luc: I felt like they were more technical questions about how to approach the program. Maybe, yeah, I don't know, maybe I was expecting something else from a psychologist.*
		Superficial	*Sophie: I don't feel like I was seeing a therapist for in‐depth work during that time.*
		Restrictive framework	*René: She works within the framework that has been set for her, and this does not in any way call into question her qualities, but it seems to me that things are being formatted rather than exchanged between two people on points that might bother me or questions I might have.*
		Filling up forms	*Françoise: I told her about my difficulties. Well, she understood, there was no problem, but then, she couldn't really give me any other solutions because I had to follow the program, so if you want, she was doing her job, she was filling in her data, she needed to collect data.*
	Emotion	Guilt‐inducing	*Françoise: I have a personality that makes me, I think I beat myself up enough already (laughs), so having someone else, a professional, tell me, “You didn't respect the rules,” well, yes, thank you, I know I didn't respect them (laughs). It was already bothering me enough.*
Areas for improvement	Change the frequency of exchanges	Every week would be better	*Hélène: Maybe a study like that would work better for me (…) with regular exchanges (…) once a week.*
		Every week would be too much emphasis	*Olivier: I think that's enough, not to have any more, so as not to focus everything on that. That's what I think, I don't know, for example, if there had been one session a week with the psychologist, I think that might have been too much, because it would have given too much importance to it.*
		Every other week	*Isabelle: I don't know, maybe every other week? We're in the thick of it, we don't have time to forget about it. Anyway, we're sticking to our goal, our program, yeah, every other week could have been better, maybe, I don't know.*
		Avoid relapses with a review after a few months	*Michel: The only concern I have is that once the program is over (…) maybe it would be worth having a little feedback, I don't know, after three or 6 months, a bit like now. (…) A quick chat to see if things are still going well, maybe to make sure that everything is validated, that we're doing well, uh… That we're continuing to… That there's no relapse, so to speak.*
	Other means of communication	Face‐to‐face	*Pascale: Direct physical contact, perhaps at least once at the beginning. It would have been, perhaps… Well, I would have appreciated that kind of interaction.*
		Visio	*Stéphane: [Video conferencing] would have been a better compromise, yeah. You see, when you're on the phone, you can pretend. It's terrible what I'm saying, isn't it? When you see a therapist, you say, “Are you okay?” “Yes, I'm fine, everything's fine,” and so on. But when you're on video chat, you can see the person, so it's harder to pretend!*
	Content	Adaptation	*Françoise: If someone had said to me, “It's okay, you'll adapt, don't worry,” maybe I would have handled it better.*
		Hypnosis, acupuncture, breathing techniques	*Hélène: Hypnosis or acupuncture or I don't know. Well, anything that can have a beneficial effect on sleep or what's it called? Breathing techniques. (…) So, well, it could be techniques like that that might help a little more.*
		Cognitive restructuring to be addressed further	*Hélène: Regarding the grids there (…) I might have needed a little more explanation on that.*
		Digging deepr	*Emma: Something a little more personalized where we ask questions about causes that are a little more psychological. Yes, a bit like a therapy session, more traditional therapy. Um… A psychotherapy session. To dig a little deeper into the why, perhaps.*
		Discussing other topics	*Bernard: There wasn't much exploration of the causes, questions about other things that could be weighing on me, like pain and all that. And anxieties, worries. It wasn't something she did. I think maybe I needed more than that.*
	Professional	Non‐psychological follow‐up	*Audrey: So whether it's a psychologist or not who's following us, I think it's okay… At least for me, it wouldn't have changed anything. It's just knowing that there's someone who's following us in the program. Whether it's someone focused on psychology… Well, anyway, it doesn't matter. For me, at least, it wasn't a plus.*

#### 3.1.1 Theme 1: Coping With the Program

The participants encountered difficulties. They all mentioned the impact of cancer and its treatments on sleep. These difficulties can be exacerbated when implementing the program due to material, contextual, relational, and psychological factors. Participants developed coping strategies to complete the program successfully.

##### 3.1.1.1 Difficulties due to Physiological and Psychological Factors

Patients often cited symptoms of cancer, unpleasant consequences of treatments, and pain as reasons for sleep disturbances. These symptoms acted as an unwelcome reminder of the illness when patients wanted to move away from it. Additionally, long‐standing sleep difficulties appeared more challenging to address. Patients expressed forms of anxiety (panic attacks, anticipatory anxiety, and rumination). The program itself could generate performance anxiety and guilt for not applying all taught rules properly. Some patients associated sleep with death, which represented an additional difficulty.

##### 3.1.1.2 Difficulties due to External Factors

Material conditions appeared to be obstacles, such as having a small room that limited time out of bed except for sleep, a constantly changing schedule, or frequent relocations. When life is unsettled, adjusting a sleep routine can be difficult. In this respect, patients who completed the program during holidays faced several challenges. For example, travel often involved time differences, and waking up early daily while not working could require too much effort for some. Conversely, returning to work could also create tension and worsen sleep disturbances. Family life could also be an obstacle, as some feared disturbing relatives by getting up and making noise.

##### 3.1.1.3 Difficulties Related to the Program

Initially, the program could seem too abstract for some. Several patients had high expectations they later realized were unrealistic. Sleep restriction was challenging, especially waiting to go to bed while exhausted. Some struggled to avoid naps due to cancer‐related fatigue. Sleep hygiene rules—avoiding coffee, limiting screens before bed, removing the television, and not reading in bed—were difficult, particularly since patients noticed the effort more than the benefits. Getting out of bed when unable to sleep was often the hardest rule.

Two aspects were especially complex: maintaining a consistent sleep schedule and completing cognitive restructuring modules, which many found difficult to understand and apply.

##### 3.1.1.4 Coping Strategies

CBT‐I is challenging to implement. To manage this, patients developed strategies to follow the rules more easily, such as moving to avoid falling asleep too early, setting alarms to limit phone use, staying active during nighttime awakenings, and focusing on the rationale behind the rules. Because the sleep diary was difficult, they also found ways to cope with memory issues and better use the tool.

Cognitive restructuring was frequently mentioned. Patients repeated modules, created paper versions of Beck's columns, and developed techniques to counter negative thoughts and promote positive ones, including breathing exercises, movement, hobbies, and repeating “I think of nothing” to occupy their minds.

Many sought to maintain long‐term benefits and prevent relapse by revisiting the program, summarizing and printing the rules, displaying them, and downloading the videos.

##### 3.1.1.5 Remaining Difficulties

The same problems encountered during the program continued after it ended. The disease, its treatments, and their consequences, as well as untreated pain and fatigue, were potential causes of sleep disturbances for some patients. Of the tools proposed by the program, one was still tough to implement: getting up at night. Also, cognitive restructuring remained impossible for some patients due to overwhelming thoughts and emotions.

##### 3.1.1.6 Post‐Program Needs

The primary need was to further improve sleep. Some patients were willing to take sleeping pills or melatonin, while others preferred to stop anxiolytics and avoid relying on external aids to fall asleep. One patient still struggled to recognize signs of sleep and respond appropriately. Several mentioned consulting specialists to assess sleep patterns or manage treatment side effects.

Regarding the program, some retained the information, while others would have preferred written summaries or video access. Few wished to continue calculating sleep efficiency.

Psychologically, several wanted to pursue cognitive restructuring and address cognitive biases, and a few recognized the need to begin psychotherapy.

#### 3.1.2 Theme 2: Connections at Play

All patients emphasized the importance of the connections between them and HCP, their family, other patients, and their sleep difficulties.

##### 3.1.2.1 Healthcare Relationships

Many patients expressed gratitude for receiving treatment at such high‐quality hospitals and for being included in this research. However, this can also be seen as a form of relief and compensation, as they had waited a long time to have their sleep disorders treated. Some had felt misunderstood by their general practitioner or expressed distress about feeling lonely, especially after their treatment ended. Additionally, several patients felt that their oncologist did not have sufficient time to inform them about alternative treatments. Patients needed to be proactive to find solutions among the hospitals' offerings.

##### 3.1.2.2 Family Ties

Many patients discussed how the program affected their family life. They feared that following the program could disturb their relatives' sleep. Some patients received support from their families in the form of reminders about sleep hygiene rules, company at night, and participation in the program. If their loved ones did not voluntarily participate in the program with them, some patients attempted to teach them the principles. Others, however, expressed difficulties with their family. Indeed, the program required patients to establish a different sleep schedule from their relatives. Rarely, patients were blamed for their sleep patterns and behavior.

##### 3.1.2.3 Peer Interactions

Patients often expressed feeling alone in their struggle with cancer and sleep disorders. Furthermore, regardless of whether they were satisfied or disappointed with the program's effects, many of them wanted to make this type of treatment available to as many people as possible, including their insomniac friends, other hospital patients through other professionals, and general practitioners. Everyone should have access to it. According to some patients, the best way to spread the word might be through patients themselves.

##### 3.1.2.4 Reconceptualizing Sleep

Many patients developed a new perspective on sleep. They no longer feared sleep or fought against it. They stopped trying to sleep more and used the program's tools to view sleep more objectively. They realized they had preconceived ideas about sleep. Sleep was no longer a problem, but rather something you thought about when difficulties arose, without paying too much attention to it. When insomnia struck, nights were no longer unpleasant but were viewed differently. Several patients had learned to accept that some nights would be less restful than others, and they had come to terms with this idea.

Thanks to the program, many patients reduced their anxiety mechanisms. Some no longer compared their current sleep to their previous sleep, did not ruminate before sleep, and looked forward to their days in a much more positive way, despite occasional sleep deprivation. Overall, the program helped them stop over‐interpreting their difficulties and see them as normal. Similarly, the program enabled some patients to change their self‐perception, encouraging them to be less demanding and to stop feeling guilty.

#### 3.1.3 Theme 3: Benefits and Limits of the Psychological Guidance

Patients were able to share their thoughts on the psychologists who supported them through three to four CBT phone calls during the program. They praised the psychologists and identified the many ways in which they were helped. While many patients found the support helpful and effective, a few found it limited or useless.

##### 3.1.3.1 A Human Relationship

Nearly all patients found their psychologist to be pleasant and praised their human qualities. Patients also appreciated their availability and reassuring presence. They emphasized how this added a human touch to the program, thanks to the psychologist's listening skills. A few people also highlighted the value of having an outside perspective. Finally, many patients appreciated the phone calls, as they would have felt more alone without them.

##### 3.1.3.2 Numerous Roles

Several roles have been identified. The psychological interviews, with their predefined regularity, provided a helpful framework that almost all patients appreciated. This regularity of exchanges also enabled assessments. For some patients, the first interview was the most important because the psychologist helped set up the program and define or redefine the objectives. Many patients also highlighted the educational value of the psychological guidance, which clarified complicated modules, such as cognitive restructuring, and taught proper tool usage. Additionally, psychologists adapted the program to participants' personal situations as much as possible. When necessary, they suggested further psychological follow‐up.

##### 3.1.3.3 Supportive Guidance

Psychological guidance was generally appreciated. Participants often described it as important, effective, and useful. It was beneficial in allowing them to express their feelings. On a cognitive level, the interviews encouraged patients to reflect and ask themselves new questions. Discussions with psychologists helped to maintain motivation, particularly thanks to the regularity of the exchanges. Several patients expressed that this helped reduce their performance anxiety on an emotional level. They were thus able to stop feeling guilty.

##### 3.1.3.4 A Limited Support

Several participants were disappointed with the level of support they received, which did not align with their expectations of psychotherapy. Some found the discussions useless, unnecessary, and unhelpful, especially if they were already receiving psychotherapy elsewhere. The main criticism was that the discussions felt superficial and overly technical, and that the research framework was too restrictive, consisting more of checking boxes than facilitating mental development. Rarely did the psychologists' feedback elicit negative emotions, such as guilt.

##### 3.1.3.5 Areas for Improvement

While most patients were satisfied with the support provided, some participants discussed the frequency of calls. Some felt that one call per week would be preferable, while others thought that every other week would be better. Nevertheless, a few patients emphasized the importance of not scheduling too many calls, lest the focus become solely on sleep disorders. One patient concluded from the research interview that conducting this type of evaluation 3–6 months after the initial assessment would help maintain the program and prevent relapses.

Several patients also reported needing eye contact, at least initially, to facilitate communication. While they mentioned in‐person care, video communication would also have been an acceptable alternative. In terms of content, some would have liked to see a little more flexibility. Other techniques, such as hypnosis or breathing exercises, were rarely requested. Cognitive restructuring, which posed a problem for many patients, would need to be addressed further during discussions with psychologists. Several patients would like to see more discussion of the psychological causes of sleep difficulties, as well as other topics such as pain and anxiety. Finally, a few patients suggested that guidance could be provided by a professional other than a psychologist, as the essential was the guidance itself, not the type of professional.

## 4 Discussion

This study aimed to explore the experiences of patients who underwent ICBT‐I with professional phone guidance. The study sought to explore patients' experiences of the program, with a focus on their perception of the professional guidance.

Most patients appreciated ICBT‐I with professional guidance, even when they did not achieve the initially desired results. Nevertheless, some patients encountered difficulties during the program. In addition to the difficulty of making specific behavioral changes, they experienced physiological problems (pain, treatments, and illness), psychological difficulties (anxiety), and difficulties related to external factors (work, vacations, and family). Therefore, it is essential to pay close attention to these factors when including patients in this program in the future, to help them better cope with these potential difficulties. It may also be necessary to maximize the effects of this type of intervention based on patients' trajectories and stages of care. Participants expressed that when returning to work or going on vacation is not an ideal timing for them. In Shaffer et al.'s study [15], participants identified the best moment to focus on sleep after treatment ended. Participants in our study said that they felt abandoned when their treatment ended and that remote care would facilitate a smoother transition.

Participants experiencing these difficulties viewed the program as a threat and an additional stressor related to their illness. Although they were not the majority of patients in our sample, their perspective must be considered. ICBT‐I is indeed an intensive treatment that requires effort from patients. Following Lazarus and Folkman's theory [16], providing psychological guidance to patients who have difficulty coping with the program can transform what is viewed as a threat into a challenge. This can be done by offering problem‐solving and adaptation strategies, helping patients identify social support resources, and emphasizing the importance of behavioral changes through psychoeducation. These strategies appeared to be effective at managing sleep disorders in patients with colorectal cancer [17]. Further research on coping strategies in relation to sleep and cancer is warranted.

Patients often expressed their desire to inform other patients. This fulfills one of the needs identified by Stern et al. [18] in remote care: a sense of belonging to a group. Patients also need to receive information regarding their disease and treatments at the hospital, sometimes highlighting shortcomings in this regard. Patients have consistently expressed the need for information in oncology [19]. Better information is actually what patients expect when digital care is implemented [20]. Therefore, institutions still need to improve their institutional communication, whether during medical consultations or digital care.

Additionally, some participants reported a lack of support from their families. Conversely, the support they received may have helped them implement the necessary changes. Indeed, some authors recommend including family members in ICBT‐I to maximize therapy's impact and improve treatment adherence [21].

Patients identified many roles for psychologists, and they often appreciated their help. Even when the interviews were not particularly helpful, having an appointment led to greater motivation and involvement on the part of the participants. This likely demonstrates the strength of the therapeutic alliance established in the first guidance session. In CBT, this alliance is partly based on setting goals, reporting back to a professional, and receiving help to implement new behaviors.

Nevertheless, several people questioned the value of psychological guidance. Interestingly, the qualities that some patients praised (adaptability, humanity, and listening skills) were precisely the qualities that other participants felt were lacking, despite being treated by the same professionals. This echoes the needs identified in the Digital Therapeutic Alliance [18]: the ability to express oneself and receive personalized responses. Several patients also emphasized the importance of human connection. This aligns with the discussions in focus groups of HCP involved in this program [22]: virtual remote interventions should complement, rather than replace, human connection [23].

## 5 Limitations

Several patients emphasized that they were aware of participating in a research study. While this encouraged some to adhere to the treatment, this knowledge influenced their perception of the treatment, sometimes encouraging their adherence, other times leading them to interpret the psychologist's attitude as being guided by a research objective rather than a clinical one.

In addition, our sample consisted mainly of women with breast cancer. While this provided deep insight into this specific population, it may limit the transferability of the findings to patients with other types of cancer or different demographic profiles.

## 6 Strengths

A major strength of this study is its conduct in real‐life conditions with minimal exclusion criteria, involving a diverse group of 48 patients from three different regions. This sample size provided high information power, allowing for a robust and detailed developed codebook. The inclusion of patients with varied profiles and program outcomes ensured a rich diversity of perspectives across the groups. Furthermore, using a codebook approach facilitated a systematic team‐based analysis, enhancing the clinical utility of the findings for improving the program's application in hospital settings.

## 7 Clinical Implications

- Pay attention to people experiencing difficulties based on characteristics such as the impact of the disease and treatments, expression of pain and anxiety, relationship difficulties, and long‐lasting sleep disorders (before cancer onset), as well as the timing of personal care, such as discontinuation or resumption of treatment, transition periods, and return to work. Investigate these difficulties with each individual to determine if they are ready to begin CBT.
- Work with each person to make their expectations more realistic, thus promoting a sense of self‐efficacy.
- Encourage strategies that focus on adapting techniques, seeking social support and emphasizing the meaning of behavioral changes through psychoeducation. Help patients to identify this support in their social circle. This will help shift the perception of the program from a threat to a challenge to be overcome.
- In a clinical care setting, investigate and respond to patients' informational needs. Develop effective communication and informational strategies at institutional and interpersonal levels.
- Define the frequency and mode of communication with patients (phone or video calls) based on their needs at the start of treatment.

## 8 Research Implications

- Research on coping strategies related to sleep and cancer together would be helpful.
- Future research should consider conducting joint interviews with HCP and patients to define and implement the most effective methods for communicating and sharing important information.

## 9 Conclusion

The qualitative component of Sleep‐4‐All‐2.0 enabled paying close attention to patient feedback about their experience with the Insomnet program. Participants were generally delighted with the treatment. They expressed a desire to connect with other patients, and they sought support and information from HCP. Psychological guidance was important for practical reasons (implementation, development, and consolidation of the modules) and emotional reasons (feeling less alone, diminishing performance anxiety, and persisting with the program).

## Author Contributions

J.J. (the research engineer) recruited the participants, led the focus groups, analyzed the data, and wrote the first draft of the manuscript. L.Z. and D.B. (the principal investigator) contributed to the recruitment process. A.L.S analyzed half of the data. J.J., A.L.S., and D.B., all trained in qualitative analysis, developed the codebook progressively and discussed it several times during the analysis. D.H. and A.M. provided all professional guidance. D.B., C.C., S.D., and J.S. contributed to the conception and design of the study. E.G. and G.P.B. (study co‐investigators) contributed to the study implementation in their cancer center. All authors read and approved the final manuscript.

## Conflicts of Interest

The authors declare no conflicts of interest.

## Supporting information


Supporting Information S1

Supporting Information S2

**Table S1:** Theme 4 – Patient’s recommendations.

## Data Availability

The data that support the findings of this study are available from the corresponding author upon reasonable request.
