# Supplementary material for: Exploring the Experiences of Cancer Patients Following an Internet‐Based Cognitive Behavioral Therapy for Insomnia With Professional Phone Guidance: The Sleep‐4‐All‐2.0 Study
Source: Psychooncology. 2026 Jun 26;35(7):e70532. doi: 10.1002/pon.70532 (PMC13309683; doi:10.1002/pon.70532)
Supplement: Supplementary file 1 — Supporting Information S1 [file PON-35-e70532-s001.docx]

Supplemental file

Theme: Patients' Recommendations

When asked how the program could be improved, many patients said it was perfect the way it was. However, others offered ideas to enhance the experience of future patients.

*Timing*

A couple of patients raised the question of the right timing regarding treatment and vacation. A few people said that being freed from treatment-related apprehensions would be desirable. Vacations, on the other hand, did not receive unanimous approval. While some thought a vacation would be a good time to devote to the program, others, as mentioned above, did not want to make the effort and get up early during that time.

*Form*

Many patients expressed a desire for the program to be available as a phone or tablet app rather than a less user-friendly web version. Additionally, some wanted improvements to the appearance of the text and information, making it easier to read. Finally, many wanted an easy-to-print summary so they could study the program without using a computer. One person also requested a reminder email a few months later to help him overcome his difficulties and keep him engaged with the sleep hygiene rules.

*Content*

In terms of content, many patients commented on the diary and cognitive restructuring exercises. For example, one person would have preferred to receive text messages for filling out the diary rather than reminder emails. Some found the diary limited and incomplete. Some people wished they could specify the quality of their sleep and add information of their choice. Some patients wanted to keep access to the diary after the program ended, while others preferred not to have a diary at all.

As for cognitive restructuring, it would require more time, modules, and illustrative video examples to facilitate understanding and integration.

*Handling Related Issues*

Many patients reported that it was difficult, if not impossible, for them to sleep when they were in pain. Thus, pain appeared to be an obstacle to better sleep that needs to be alleviated to improve nights. Similarly, some patients perceived their pathologies as being associated with their sleep disorders and wanted to be treated holistically.

*Adaptation and Support*

In addition to the psychological interviews that will be discussed later, some people required technical support at the beginning of the program to help them understand the tool. This should be implemented systematically. Furthermore, some patients would like to see their individuality taken into greater account and would like the program to be adapted more closely to their personal situation.

*Fostering Human Connections*

Although most patients enjoyed the cartoons, they sometimes had difficulty identifying with the characters. For example, some patients would have liked to see testimonials from real patients to learn about their experiences with the program. Some people would have preferred a group modality with classic CBT. The idea of connecting with others was consistently mentioned as a way to feel less alone. This idea was discussed extensively by our sample and is therefore a theme in its own right.
