# Supplementary material for: Exploring the Experiences of Cancer Patients Following an Internet‐Based Cognitive Behavioral Therapy for Insomnia With Professional Phone Guidance: The Sleep‐4‐All‐2.0 Study
Source: Psychooncology. 2026 Jun 26;35(7):e70532. doi: 10.1002/pon.70532 (PMC13309683; doi:10.1002/pon.70532)
Supplement: Supplementary file 2 — Supporting Information S2 [file PON-35-e70532-s002.docx]

Appendix 1. Presentation at the beginning of each phone call

You followed the Insomnet program for 6 to 12 weeks as part of the Sleep-4-All-2.0 study. I'm the research engineer responsible for conducting the interviews. The purpose of these interviews is to gather feedback from Insomnet participants. So I'll be asking you questions about how the program went for you, what you thought of it, and what you're taking away from the experience today. We'll need about half an hour.

Every point of view is important, so please feel free to express your personal perspective; there is no right or wrong answer. Your comments will remain confidential and anonymous, and will not be shared with the professional who accompanied you during the program. For the purposes of the study, our interview is recorded. It will be transcribed in such a way that you cannot be identified.

Do you still agree to this interview? Do you have any questions?

Appendix 2. Questions

1. Could you first tell me what motivated your participation in the Insomnet program? What were your expectations? Were your expectations met?

2. Tell me how the program went for you?

2.1 What did you like best/least?

2.2 What worked best/least/not at all for you?

2.3 What difficulties, if any, did you encounter?

2.4 What motivated you to continue the program over the long term?

3. How did your sleep evolve during the program? And since you completed the program? Do you feel that sleep is still a problem for you?

4. What do you still need from the program? Are you still using any of the tips or tools?

5. What role did the resource person play for you during the program? [If necessary, specify: Were these exchanges helpful? To what extent? How did you prefer to communicate - phone/email]. Would you have needed or wanted another form of support?

6. What advice would you give us to improve the program?

7. Do you have any comments or thoughts to share on the user experience of the program? Have you encountered any bugs or technical difficulties? Have you accessed the program using a computer, tablet, or telephone?

8. Anything else you'd like to add?
