# Supplementary material for: Exploring the Experiences of Cancer Patients Following an Internet‐Based Cognitive Behavioral Therapy for Insomnia With Professional Phone Guidance: The Sleep‐4‐All‐2.0 Study
Source: Psychooncology. 2026 Jun 26;35(7):e70532. doi: 10.1002/pon.70532 (PMC13309683; doi:10.1002/pon.70532)
Supplement: Supplementary file 3 — Table S1: Theme 4 – Patient’s recommendations. [file PON-35-e70532-s003.docx]

Table S1: Theme 4 – Patient’s recommendations

| Recommendations | Timing | On treatment |  | *Sandrine: But I don't know whether this study is intended for any patient, whether still on treatment or no longer on treatment (...) For me, a person no longer on treatment perhaps doesn't have the same apprehensions as someone on treatment.* |
| --- | --- | --- | --- | --- |
|  |  | Vacation |  | *Patrick: The question I was asking myself was (...) should there be a vacation during this, during this program to have time. To devote a little... Well, to devote a little time.* |
|  | Form | Turning it into an App |  | *Céline: To be able to make (...) an application (...). I think it would be more practical on a phone or a tablet. When you go on vacation, for example, and you're in the middle of a program, well, it's not always easy to have a computer at hand to do it.* |
|  |  | A clearer text |  | *Martine: Do the text differently. Pass it off as a kind of PowerPoint (...) It looks playful, but there's still a lot of information in there (...) There was a bit too much text (...) The layout of the presentation is not at all optimal.* |
|  |  | Fighting oblivion | Post-program reminder e-mail | *Olivier: Maybe two, three months into the program, it could be a little reminder, a little... But even by e-mail, a little reminder e-mail: well, think again about taking another look at the... At the whole program, to see if there are any weak points.* |
|  |  |  | A synthesis of the program | *Nathalie: What I would have liked (...) is to have a written trace, to be able to, uh rather than have everything on computer, there are certain things I would have liked to have on paper, to have them with me from time to time, and to be able to remember them. But now, as it's on the computer (...) I tend not to do that.* |
|  | Content | Sleep diary | Text message reminder | *Isabelle: Answering by phone would have been easier for me, there may have been days when I didn't answer or I would have answered if it had been on my phone. (...) A short text message: “Hello, how did your night go?”, ask a couple of questions would have been easier for me.* |
|  |  |  | Free text | *Martine: And also, but then it's difficult, the way of filling in [the diary]. It was... Well, it didn't cover all the possibilities, but that's difficult too. But at... At the time, the idea was that there were things in my life and in my nights that weren't covered by this questionnaire. For example, what woke you up? You see, pain is important. You've got to... Well, you've got to, you've got to, you've got to leave a little tab for free text. Because if the guy tells you: “I woke up at two o'clock because I was anxious”. It's not the same as saying: “I woke up at two o'clock because I was in pain”.* |
|  |  |  | Sleep quality index | *Marine: So for me, it's really about, um, having a quality index for, um, sleep. Because I find that, in the end, it's more the quantity that's... That's calculated.* |
|  |  |  | Post-program access | *Laure: I like this application, where you can see how many hours you've slept and so on, and all that, how it's calculated, the quality of sleep, and so on. And I think it could be good on uh... On the months after, that people who have followed the study, can uh... continue to do so if they wish to do so in fact.* |
|  |  |  | Not having a sleep diary | *Bernard: For me it's better not to do a sleep diary every single day. The more I think about sleep, the less good it is.* |
|  |  | Cognitive restructuring | Adding videos | *Marine: I think that part about automatic thoughts, well... In fact, they were concepts that, er... I don't know, maybe they're concepts that we're not used to. I had to go over it again and again to get it to speak to me. So I don't know if it's possible to explain it differently, I don't know, uh... Or maybe uh... I don't know, use video examples each time, or examples that are a bit more uh... I... Well, that part was really hard for me to get my head around. I had, I had trouble understanding it (laughs).* |
|  |  |  | Deepening cognitive work | *Maurice: Dig deeper into the question of thoughts, bad thoughts or harmful thoughts* |
|  |  |  |  | *Marine: Everything with the cognitive grid, uh, I found that one a bit heavy. (Laughs) I don't know if it should be put in several parts. There are parts in text, and I found it a bit... A bit difficult to... To integrate.* |
|  | Handling related issues | Sleep and pain |  | *Alain: Maybe something a little more focused on pain. Because it's, it's a bit my, my case at the moment I... I have little pains that work on me at night. So uh, I think there should perhaps be some, some techniques a bit of... So not, not to make the pain go away because it's not, it's, it's not the goal but uh, the link between... Between sleep and pain.* |
|  |  | Treating related pathologies |  | *Stéphane: And above all, um, treat related pathologies, because if you've got a, a guy, I'll give you an image, who's a cripple, and you ask him to do, to prepare for a four-hundred-meter race, no, there's a problem. (...) And there's bound to be a gap between the person's condition and the audience you're addressing. You have to address an audience that's, in quotation marks, uh, without any aggravating pathology, and that's able to receive what you're distilling.* |
|  | Adaptation and support | Technical support at the start |  | *Laure: You kind of need to be accompanied, accompanied in fact at the beginning. And uh... I'm used to computers and all that, but I think for other people it must be a bit more complicated.* |
|  |  | Adapting the program |  | *Alain: But I, I think it would be worthwhile to, to have something... Well, something a bit individualized.* |
|  | Fostering human connections | Testimonies from real patients |  | *François: Well, maybe get people to testify. Real people. (...) If people could talk about their difficulties, we wouldn't feel alone. And we'd see people who have the same difficulties as us. (...) For me, that would have been an additional motivating factor.* |
|  |  | Feedback |  | *Sylvie: And then yes have a little feedback on how it went with the other people.* |
|  |  | Group intervention |  | *Françoise: Maybe set up groups with a doctor who leads the group and gives advice, where you realize that you're not alone in your situation and that sometimes people have tips and tricks that can help you, because you're pretty much on your own.* |
